# Dataset of protein species from human liver

**DOI:** 10.1016/j.dib.2017.04.051

**Published:** 2017-05-04

**Authors:** Stanislav Naryzhny, Maria Maynskova, Victor Zgoda, Alexander Archakov

**Affiliations:** a Orekhovich Institute of Biomedical Chemistry of Russian Academy of Medical Sciences, Pogodinskaya 10, Moscow 119121, Russia; b B.P. Konstantinov Petersburg Nuclear Physics Institute, National Research Center "Kurchatov Institute", 1 Orlova roscha, Gatchina, Leningrad Region 188300, Russia

## Abstract

This article contains data related to the research article entitled “Zipf׳s law in proteomics” (Naryzhny et al., 2017) [1]. The protein composition in the human liver or hepatocarcinoma (HepG2) cells extracts was estimated using a filter-aided sample preparation (FASP) protocol. The protein species/proteoform composition in the human liver was determined by two-dimensional electrophoresis (2-DE) followed by Electrospray Ionization Liquid Chromatography-Tandem Mass Spectrometry (ESI LC-MS/MS). In the case of two-dimensional electrophoresis (2-DE), the gel was stained with Coomassie Brilliant Blue R350, and image analysis was performed with ImageMaster 2D Platinum software (GE Healthcare). The 96 sections in the 2D gel were selected and cut for subsequent ESI LC-MS/MS and protein identification. If the same protein was detected in different sections, it was considered to exist as different protein species/proteoforms. A list of human liver proteoforms detected in this way is presented.

**Specifications Table**

**Table:** 

Subject area	Biology
More specific subject area	Proteomics
Type of data	Tables, Figure
How data was acquired	2-DE, Mass spectrometry ESI LC-MS/MS
Data format	analyzed
Experimental factors	Tissue grinding in liquid nitrogen. Protein extraction by Rabillound buffer
Experimental features	2D electrophoresis (1st dimension: pH 3–11 gradient; 2nd dimension: 12% PAGE). Cutting the gel to 96 sections. Trypsin digestion of proteins. ESI LC-MS/MS analysis of the peptides.
Data source location	The data was collected at Institute of Biomedical Chemistry, Moscow, Russia
Data accessibility	The data is with this article. It is also deposited in the Mendeley Data http://dx.doi.org/10.17632/k2rwm88v6b.
	http://dx.doi.org/10.17632/2997h4fcfz.1

**Value of the data**

• The data allow the estimation of the distribution of proteins and protein species/proteoforms in human liver cells according to their abundance.
• It is possible to easily extract information about sets of proteoforms that are encoded by the same genes and the abundance of these protein species/proteoforms as well.
• The data could be a starting point for quantitative research of protein species/proteoforms

## 1 Data

**Fig. 1:**
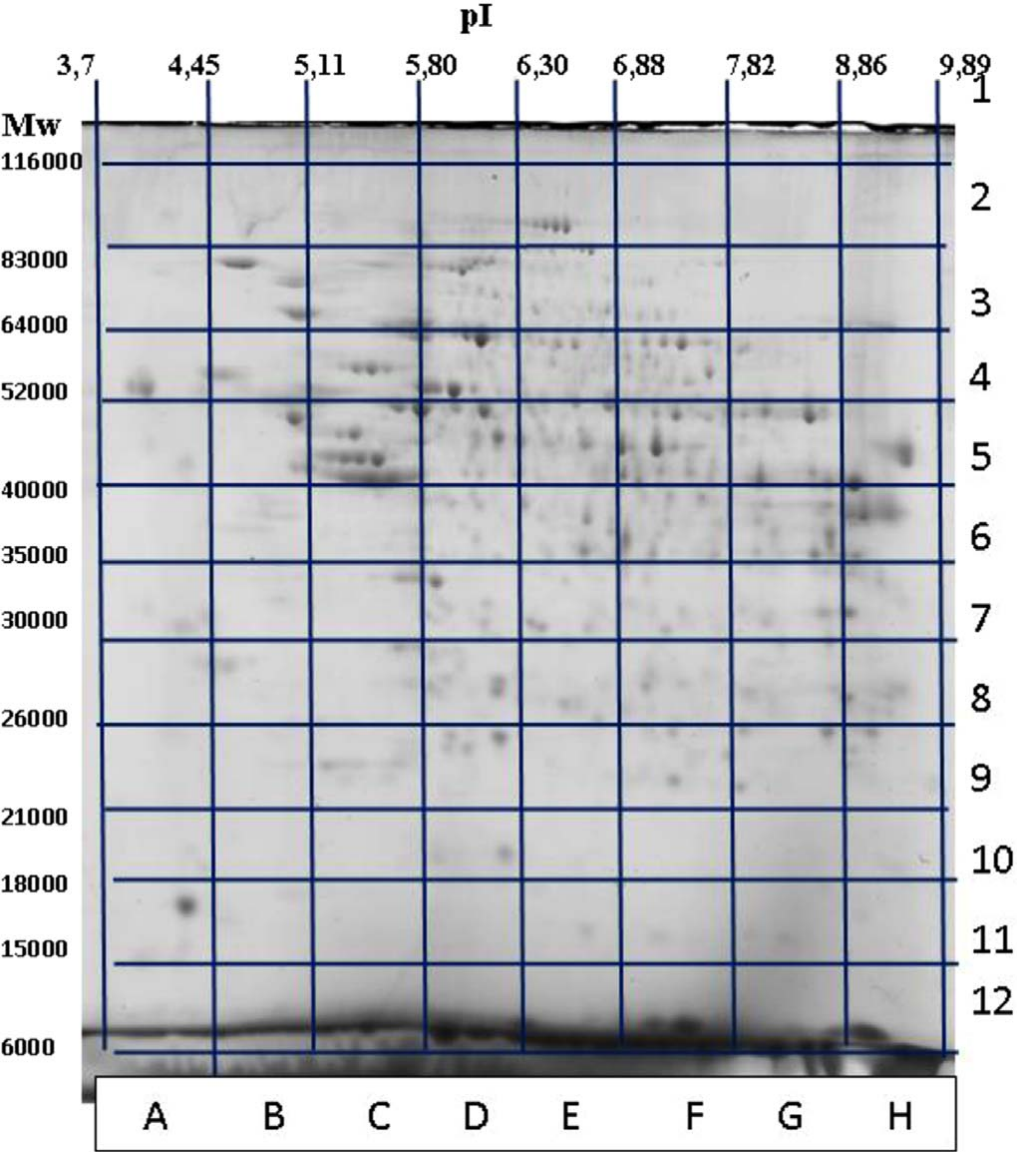
2-DE of human liver proteins. Staining was performed by Coomassie Brilliant Blue R350. Image analysis was produced using ImageMaster 2D Platinum software (GE Healthcare). The 96 sections in 2-DE gel were selected and cut for following ESI LC-MS/MS analysis [1].

The extracts of human liver or HepG2 cells were treated with trypsin using the FASP protocol. The peptides produced were analyzed by ESI LC-MS/MS. The lists of proteins detected are presented in Supplementary Table 1. The extracts of human liver tissue (300 µg of protein) were also run by 2-DE (Fig. 1). The gel produced was stained with Coomassie Brilliant Blue R350. Image analysis was performed by ImageMaster 2D Platinum software (GE Healthcare, Pittsburgh, PA, USA). Next, 96 sections were selected, given pI/Mw coordinates, and cut for subsequent ESI LC-MS/MS analysis (Fig. 1). A list of all proteins detected by Mascot (only without hemoglobin) in the human liver extracts is presented in Supplementary Table 2. Hemoglobin was removed as a major contaminant of blood plasma proteins. If the same protein was identified in different sections, it was considered to exist as different proteoforms. According to this rule, a total of 14667 proteoforms were identified.

## 2 Experimental design, materials and methods

### 2.1 Cells

Human cells (hepatocellular carcinoma (HepG2) were cultured in medium (DMEM/F12 or RPMI-1640 supplemented with 10% fetal bovine serum (FBS) and 100 U/ml penicillin) under standard conditions (5% CO_2_, 37 °C) [2], [3], [4]. To prepare samples for protein extraction, the cells were detached with 0.25% Trypsin-EDTA solution, washed 3 times with PBS, and treated with Rabillound lysis buffer (7 M urea, 2 M thiourea, 4% CHAPS, 1% DTT, 2% ampholytes, pH 3–10, protease inhibitor mixture) [2], [5]. Liver tissue samples were provided within the framework of collaboration on the Chromosome-Centric Human Proteome Project (C-HPP). Extraction was performed by lysis buffer after grinding the tissue in liquid nitrogen according to 2-DE protocol described in [6].

### 2.2 Sample preparation and 2-DE

Samples were prepared as described previously [7], [8]. Cells (~10^7^) were treated with 100 µl of lysis buffer, and proteins (~2 mg) were extracted. Proteins were separated by isoelectric focusing (IEF) using DryStrips pH 3–11, 7 cm (“GE Healthcare”) following the manufacturer׳s protocol. Samples were mixed with rehydrating buffer (7 M urea, 2 M thiourea, 2% CHAPS, 0.3% DTT, 0.5% IPG buffer, pH 3–11 NL, 0.001% bromophenol blue) in a final volume of 130 µl (300 µg of protein). Strips were passively rehydrated for 6 h at 4 °C. IEF was performed at 20 °C on an IPGphor (GE Healthcare) that was programmed to run for 24000 V-hours (Vh). After IEF, strips were soaked for 10 min in the equilibration solution (50 mM Tris, pH 6.8, 6 M urea, 2% SDS and 30% glycerol) with 1% DTT. This process was followed by a 10-min incubation in the equilibration solution containing 5% (w/v) iodoacetamide. The strips were placed on top of the 12% polyacrylamide gel of the second direction (gel size 80×90×1 mm), sealed with a hot solution of 0.5% agarose prepared in electrode buffer (25 mM Tris, pH 8.3, 200 mM glycine, 0.1% SDS), and run under denaturing conditions using the Hoefer miniVE system (GE Healthcare) at a constant power of 3 W/gel [8], [9].

### 2.3 Image analysis

Images (n=3) were analyzed using ImageMaster 2D Platinum 7.0 (GE Healthcare).

### 2.4 In-gel digestion and mass spectrometry

Gel-free sample treatment of cell or tissue lysates was performed according to the FASP assay [10]. In short, cysteines were reduced with 100 mM dithiothreitol (DTT). Excess reagent was removed by ultrafiltration in Microcon filters (YM-10) followed by a wash with washing buffer (8 M urea 100 mM Tris, pH 8.5). Cysteines were carboxyamidomethylated with 50 mM iodoacetamide (IAA), and excess reagent was removed by washing buffer followed by digestion buffer (50 mM ammonium bicarbonate, pH 8.5). The proteins were digested with trypsin (“Trypsin Gold”, 10 µg/ml, in digestion buffer) for at least 4 h at 37 °C and the resulting peptides were collected as a filtrate.

The treatment of gel pieces was performed according to the protocol described elsewhere [4], [11], [12]. Agilent HPLC system 1100 Series and columns were used (Agilent Technologies, USA). In short, the tryptic peptides were dissolved in 5% (v/v) formic acid and injected into a trap column Zorbax 300SB-C18, 5×0.3 mm. After washing (5% ACN, 0.1% formic acid), the peptides were resolved on a 150 mm×75 µm Zorbax 300SB-C18 reverse phase analytical column using a 30-min 5–60% ACN gradient in 0.1% formic acid with a flow rate of 300 nL/min. The peptides were ionized by nano-electrospray at 2.0 kV using a fused silica emitter with an internal diameter of 8 µm (New Objective, USA). MS/MS analysis was performed in duplicate using an Orbitrap Q-Exactive Plus (Thermo Scientific, USA). Mass spectra were acquired in the positive ion mode. High resolution data was acquired with a resolution of 30,000 (m/z 400) for MS and 7500 (m/z 400) for MS/MS scans. Survey MS scan was followed by MS/MS spectra of five of the most abundant precursors. For peptide fragmentation, higher energy collisional dissociation (HCD) was 35 eV, the signal threshold was 5000 for an isolation window of 2 m/z, and the first mass of HCD spectra was 100 m/z. Fragmented precursors were dynamically excluded from targeting for 90 s. Singly charged ions and ions with unassigned charge state were excluded from triggering MS/MS scans. The automatic gain control target value was regulated at 1×10^6^ with a maximum injection time of 100 ms and at 1×10^7^ with a maximum injection time of 250 ms for MS and MS/MS scans, respectively. The data were searched by Mascot 2.4.1 (www.matrixscience.com). The following parameters were applied – enzyme: trypsin, allowing cleavage before proline; maximum missed cleavages: 2; fixed modifications: carbamidomethylation of cysteine; variable modifications: oxidation of methionine, phosphorylation of serine and threonine, acetylation of lysine; precursor mass tolerance: 20 ppm; product mass tolerance: 0.01 Da. As a protein sequence database, NeXtProt (October 2014) was used. A separate decoy database was generated for the false discovery rate (FDR) evaluation. A false-positive rate of 1% was allowed for protein identification [13]. The exponentially modified form of protein abundance index (emPAI) defined as the number of identified peptides divided by the number of theoretically observable tryptic peptides for each protein was used to estimate protein abundance [14], [15].

## Figures and Tables

**Fig. S1:**
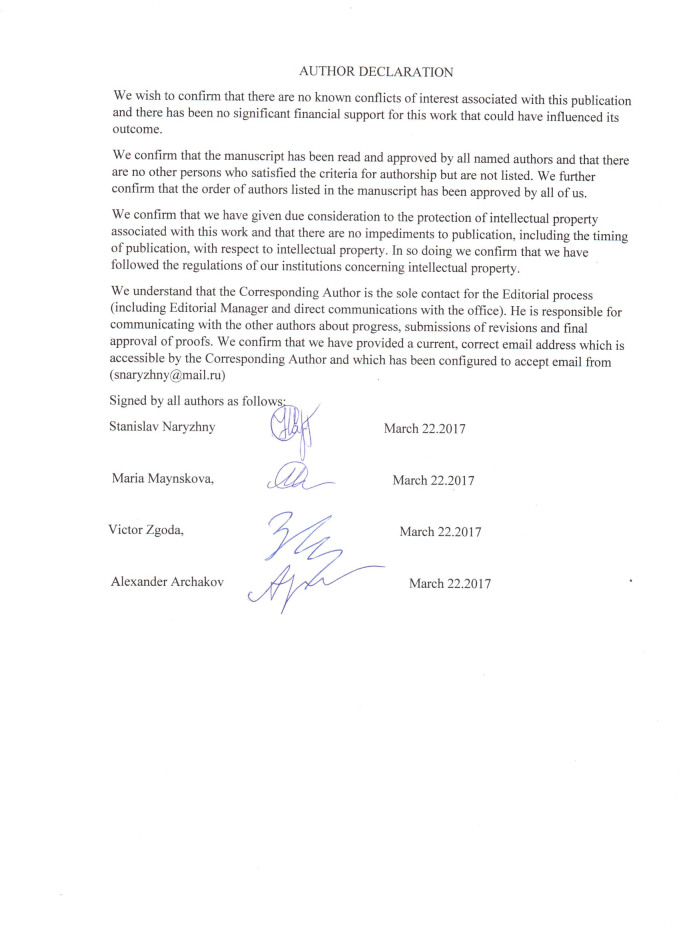
Supplementary Table 1. A list of proteins detected in extracts of the human liver or HepG2 cells by ESI LC-MS/MS using FASP protocol.

## Appendix A Supplementary material

Supplementary Table 2. A list of all detected proteoforms in the human liver extracts after 2-DE and ESI LC-MS/MS.
